# Quantifying the Transmit‐Power Threshold for Reliable Passive Detuning of Inductively Coupled Wireless Resonators in MRI: A Phantom Study

**DOI:** 10.1002/nbm.70408

**Published:** 2026-09-25

**Authors:** Ming Lu, Xiaoyu Jiang, Yajun Ma, Yurui Qian, Congyu Liao, Yang Yang, John C. Gore, Xinqiang Yan

**Affiliations:** ^1^ Vanderbilt University Institute of Imaging Science, Vanderbilt University Medical Center Nashville Tennessee USA; ^2^ Department of Radiology and Radiological Sciences Vanderbilt University Medical Center Nashville Tennessee USA; ^3^ Department of Radiology University of California San Diego California USA; ^4^ Department of Radiology and Biomedical Imaging University of California San Francisco California USA; ^5^ Department of Electrical and Computer Engineering Vanderbilt University Nashville Tennessee USA

**Keywords:** inductively coupled, passive detuning, RF safety, spatial signal ratio, wireless resonator

## Abstract

This work develops and validates a practical method for quantifying the transmit‐power threshold at which passive detuning of inductively coupled wireless resonators becomes reliable in MRI. All experiments were performed using phantoms. A near/far spatial signal ratio is computed from a series of gradient‐echo (GRE) images acquired at different nominal flip angles. This ratio reveals ineffective detuning as a characteristic L‐shaped curve whose inflection point marks the detuning threshold. The method was validated experimentally with wireless resonators incorporating different numbers of crossed diode pairs, at multiple static field strengths (3 and 7T), and with different scanner vendors. At 3T, the method produced consistent $B_{1}^{+}$ thresholds across Philips and GE scanners despite their different flip‐angle thresholds. The proposed method provides a practical, straightforward tool for acceptance testing and quality assurance of wireless resonator safety in both clinical and research MRI settings.

## Graphical abstract

Wireless MRI resonators rely on passive detuning to prevent local transmit‐field amplification. We introduce an image‐based method to quantify the transmit‐power threshold for reliable detuning using gradient‐echo images acquired over a range of flip angles. The near‐to‐far signal ratio forms an L‐shaped curve, with the turning point defining the detuning threshold. Validation at 0.55, 3, and 7 T across Siemens, Philips, and GE scanners demonstrated consistent threshold identification despite differences in hardware and transmit amplitude.

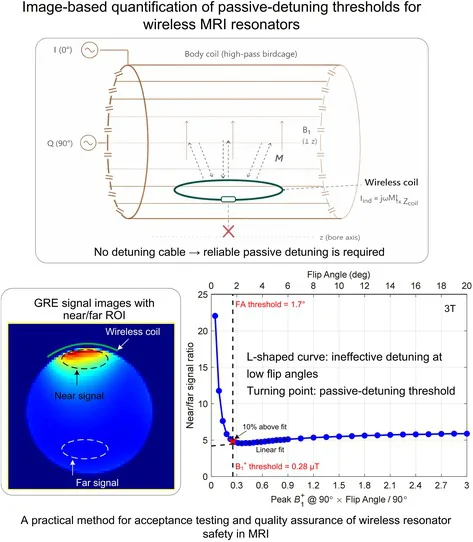

## 1 Introduction

Inductively coupled wireless resonators, also termed wireless coils or passive resonators, have a long history in MRI [1, 2, 3, 4, 5, 6]. They have been used to enhance transmit efficiency, receive sensitivity, or both, and more recently have been demonstrated in combination with multichannel local receive arrays [7, 8, 9, 10, 11, 12, 13, 14, 15, 16, 17, 18, 19, 20, 21, 22, 23, 24, 25, 26, 27, 28]. Regardless of their specific geometry or imaging application, receive‐only wireless resonators must be effectively detuned during RF transmission to avoid perturbing the primary transmit field. However, a validated method for quantifying the transmit‐power threshold at which passive detuning becomes reliable has been lacking. The present work addresses this gap by establishing a practical quality assurance and acceptance‐testing approach for evaluating passive detuning safety, distinct from prior studies focused on resonator design or SNR enhancement.

Although wireless resonators can be used to manipulate the transmit (Tx) field ($B_{1}^{+}$) in certain scenarios—for example, when the native $B_{1}^{+}$ field of the Tx coil is suboptimal [9, 10, 17, 20]—the majority of recent wireless resonator designs operate in receive‐only mode [19, 21, 22, 23, 24, 25, 26, 27, 28]. A fundamental requirement for any receive‐only resonator inside the bore of an MRI scanner is that it must be effectively transparent to the transmit RF field during excitation. Cabled receive arrays achieve this through active detuning: a DC bias is applied to PIN diodes or similar switching elements that detune each resonator during the transmit phase [29]. Wireless resonators, lacking a wired control path, instead rely on passive detuning [1, 6, 13, 14, 19].

**FIGURE 1:**
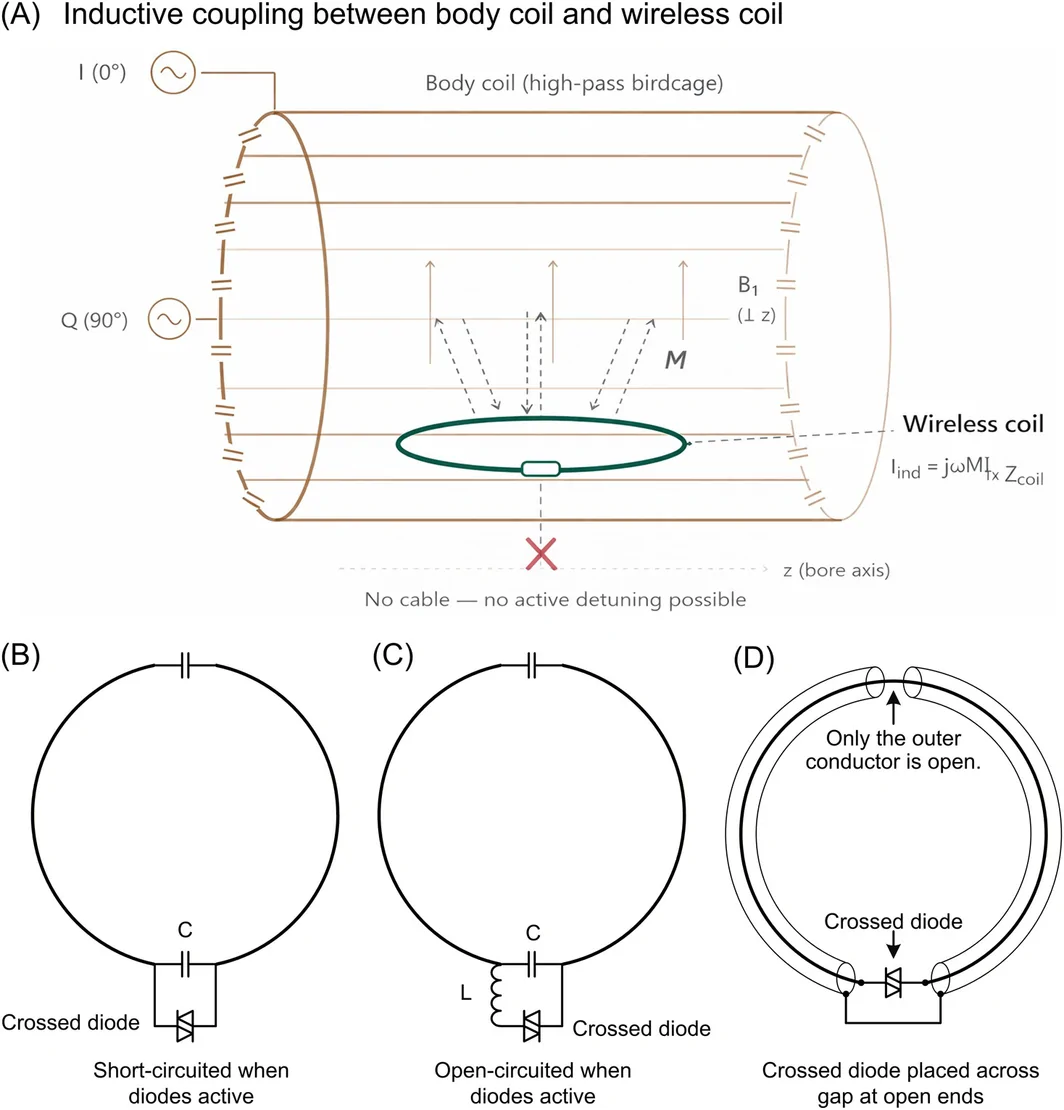
Passive detuning of wireless resonators. (A) Illustration of the induced current in the wireless resonator during RF transmission. (B) Crossed diodes in parallel with a distributed capacitor (capacitor shorted when diodes conduct). (C) Crossed diodes with a series inductor in parallel with a distributed capacitor (capacitor effectively open‐circuited when diodes conduct). (D) Coaxial coil implementation with diodes placed across the gap of the open ends. The ellipses indicate the presence of multiple distributed capacitors along the conductor.

**FIGURE 2:**
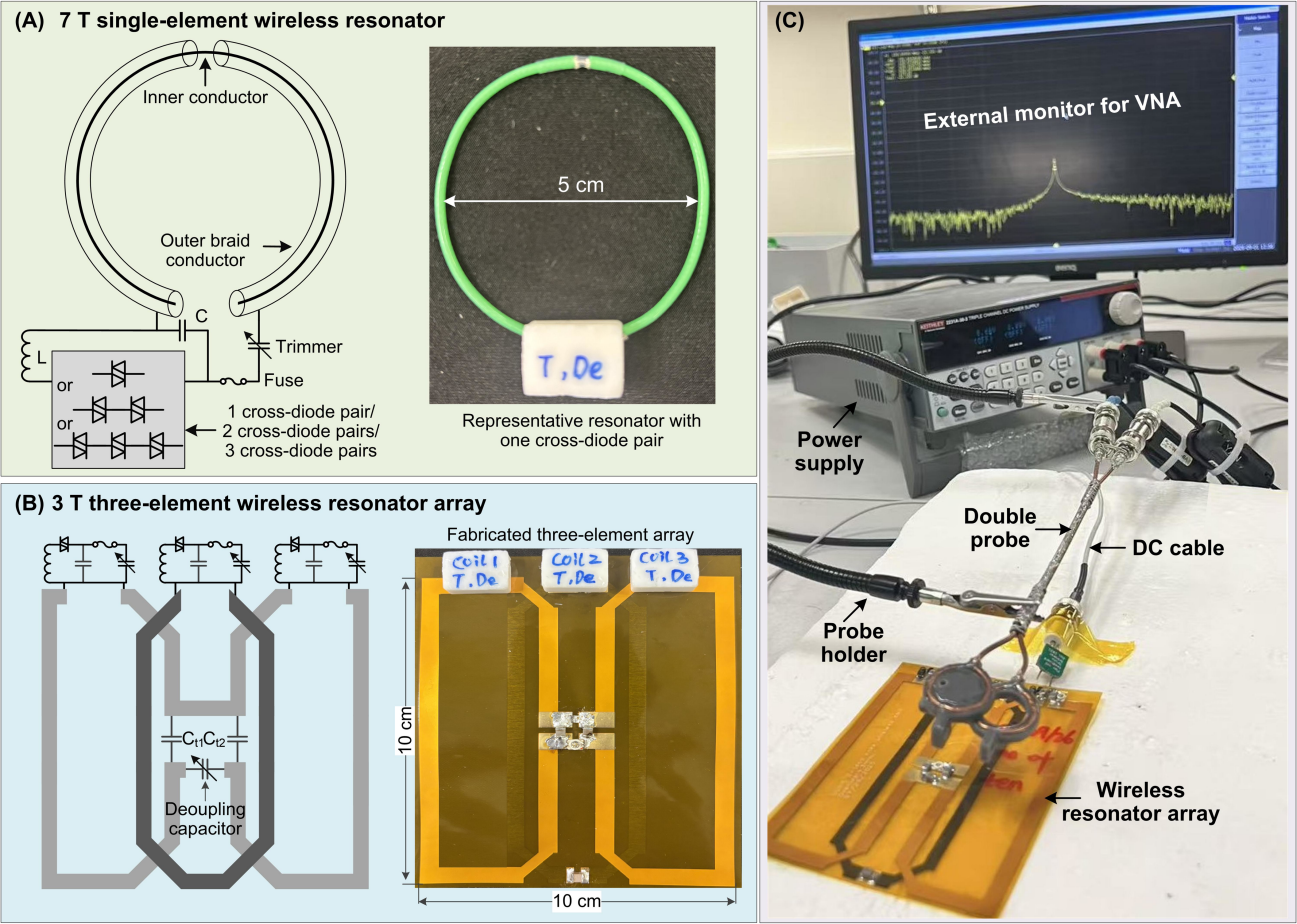
Wireless resonator designs and bench test setup. (A) Circuit schematic and representative photograph of the 7 T single‐element wireless resonator. Three resonators with one, two, or three crossed‐diode pairs were tested; the photograph shows the one‐pair version. (B) Circuit schematic and photograph of the 3 T three‐element wireless resonator array. (C) Photograph of the benchtop characterization setup. A double probe connected to a VNA was used to measure the resonance response, while a DC power supply was used to characterize the detuning response under an applied bias voltage.

In passive detuning, the mutual coupling between the Tx coil and wireless resonator induces a current along the wireless resonators (illustrated in Figure 1A). This current produces a voltage across the antiparallel (crossed) diodes that, once exceeding their forward‐bias threshold, activates the detuning circuit and deactivates the wireless resonator. Common implementations include (1) crossed diodes placed directly in parallel with a distributed capacitor so that the capacitor is shorted when the diodes conduct (Figure 1B), and (2) crossed diodes with a series inductor, together in parallel with a distributed capacitor, so that the capacitor is effectively open‐circuited when the diodes conduct (Figure 1C). These schemes can also be applied to coaxial coils, in which case the diodes are placed across the gap of the open ends rather than across a discrete capacitor (Figure 1D).

Therefore, passive detuning introduces a Tx‐RF‐power dependence that active detuning does not share. If the local RF amplitude induced in the wireless resonator is too low to forward‐bias the diodes, the resonator remains tuned and resonant during transmission. In this underdamped state, the wireless resonator can couple with the Tx coil and locally amplify the $B_{1}^{+}$ field, distorting flip‐angle uniformity and causing artifacts. It also causes the system's specific absorption rate (SAR) model to underestimate the actual local RF energy deposition. The critical question, at what Tx power passive detuning reliably engages, has not been systematically addressed. This represents an unresolved acceptance‐testing and quality‐assurance problem for receive‐only wireless resonators. As wireless resonators move toward broader biomedical and clinical application, an area of rapidly growing activity, establishing this threshold is a prerequisite for their safe translation.

$B_{1}^{+}$ mapping techniques (double‐angle method [30], Bloch–Siegert shift [31], actual flip‐angle imaging [32], and DREAM [33]), can provide direct evidence of how the $B_{1}^{+}$ field is altered by the presence of wireless resonators. Comparing $B_{1}^{+}$ maps acquired with and without the resonator confirms whether efficient detuning is achieved once the crossed diodes are conducting. Identifying the threshold at which detuning engages would require repeating the mapping over a sweep of transmit voltages. Because reliable $B_{1}^{+}$ maps require sufficient RF amplitude and SNR, this becomes least practical in the very‐low‐power regime, which is precisely where detuning failure is most likely. None of these $B_{1}^{+}$ techniques is used in the present study, as they operate in a regime in which the diodes are already conducting.

The present study focuses specifically on this passive‐detuning quality‐assurance problem, rather than on resonator‐array design, SNR optimization, spatial positioning, or in vivo imaging performance. In this work, we investigate the passive‐detuning behavior of wireless resonators at different static‐field strengths by acquiring a series of gradient‐echo (GRE) images over a range of nominal flip angles. Rather than mapping $B_{1}^{+}$ directly, we propose a complementary analysis approach that computes a near‐to‐far spatial signal ratio, $\rho_{\text{NF}}$, defined as the mean signal near the wireless resonator divided by that in a region far from it. This approach reveals ineffective detuning as a characteristic L‐shaped curve. In the low‐flip‐angle regime, the ratio is insensitive to other factors (such as receive sensitivity and receive gain) and the only quantity that can cause it to deviate from its baseline is a change in the wireless resonator's $B_{1}^{+}$ perturbation with flip angle, i.e., a failure of passive detuning. Preliminary data from this work were published as an abstract by Lu et al. [34].

We outline the simple theory behind this approach, describe the experimental protocol, and validate it across wireless resonators of different geometries at multiple static‐field strengths (3 and 7T). At 7T, we further validate the method using resonators with different numbers of crossed diode pairs and hence different passive‐detuning voltage thresholds. The method reliably distinguishes resonators with different detuning thresholds.

## 2 Theory

### 2.1 Gradient‐Echo Signal at Small Flip Angles

The steady‐state signal from a spoiled GRE sequence at spatial location r is [35]

(1) $$S(r,\alpha_{\text{nom}})=G(\alpha_{\text{nom}})\;M_{0}(r)\;C_{\text{rx}}(r)\;f\left(\alpha (r,\alpha_{\text{nom}}),E_{1}\right)\;\exp\left(-\text{TE}/T_{2}^{*}(r)\right),$$

with $\alpha_{\text{nom}}$ the nominal flip angle, $\alpha (r,\alpha_{\text{nom}})$ the actual flip angle, $M_{0}$ the equilibrium magnetization, $C_{\text{rx}}$ the receive sensitivity, $G(\alpha_{\text{nom}})$ the receiver gain (which the scanner may adjust per acquisition), and

(2) $$f(\alpha ,E_{1})=\frac{\sin\alpha \;(1-E_{1})}{1-E_{1}\cos\alpha },\;E_{1}=\exp(-\text{TR}/T_{1}).$$

At small flip angles, sinα≈α and cosα≈1, giving $f(\alpha ,E_{1})\approx \alpha$: the GRE signal becomes proportional to the actual flip angle, independent of TR and $T_{1}$ [35].

### 2.2 Effect of the Wireless Resonator on the Transmit Field

Writing $\alpha (r,\alpha_{\text{nom}})=B_{1}^{+}(r,\alpha_{\text{nom}})\;\alpha_{\text{nom}}$, we decompose the Tx efficiency as

(3) $$B_{1}^{+}(r,\alpha_{\text{nom}})=B_{1,\text{body}}^{+}(r)+\Delta B_{1,\text{wc}}^{+}(r,\alpha_{\text{nom}}),$$

where $B_{1,\text{body}}^{+}$ is the fixed contribution from the body (or local Tx) coil and $\Delta B_{1,\text{wc}}^{+}$ is the perturbation from the wireless resonator. $\Delta B_{1,\text{wc}}^{+}$ depends on $\alpha_{\text{nom}}$ through the nonlinear diode response: above the diode forward‐voltage threshold the detuning circuit activates and $\Delta B_{1,\text{wc}}^{+}\approx 0$ [13, 19], while below it the wireless resonator remains resonant and locally amplifies $B_{1}^{+}$ ($\Delta B_{1,\text{wc}}^{+}>0$). Far from the wireless resonator, $\Delta B_{1,\text{wc}}^{+}\approx 0$ regardless of $\alpha_{\text{nom}}$.

### 2.3 Spatial Signal Ratio for Threshold Detection

Let ${\text{ROI}}_{\text{near}}$ and ${\text{ROI}}_{\text{far}}$ be ROIs close to and far from the wireless resonator, respectively, and define

(4) $$\rho_{\text{NF}}(\alpha_{\text{nom}})=\frac{\left\langle S_{\text{near}}(\alpha_{\text{nom}})\right\rangle }{\left\langle S_{\text{far}}(\alpha_{\text{nom}})\right\rangle }.$$

For each flip angle, the standard error of the mean (SEM) was calculated within each ROI as the voxel‐wise standard deviation divided by the square root of the number of voxels. The uncertainty in $\rho_{\text{NF}}$ was then estimated using standard uncertainty propagation:

(5) $$\sigma_{\rho_{\text{NF}}}=\rho_{\text{NF}}\sqrt{{\left(\frac{{\text{SEM}}_{\text{near}}}{\left\langle S_{\text{near}}\right\rangle }\right)}^{2}+{\left(\frac{{\text{SEM}}_{\text{far}}}{\left\langle S_{\text{far}}\right\rangle }\right)}^{2}}.$$

The resulting uncertainties are shown as error bars in the corresponding figures.

The receiver gain $G(\alpha_{\text{nom}})$ cancels in this ratio—a practical advantage, since the scanner often adjusts G automatically when the prescribed flip angle changes. For a homogeneous phantom ($M_{0},\;T_{1},\;T_{2}^{*}$ identical in both ROIs), all tissue‐dependent terms also cancel, and Equations  (1) and  (3) yield, at small flip angles,

(6) $$\rho_{\text{NF}}(\alpha_{\text{nom}})\approx \rho_{\text{NF},0}+C\;\frac{\left\langle \Delta B_{1,\text{wc}}^{+}(\alpha_{\text{nom}})\right\rangle }{\left\langle B_{1,\text{body}}^{+,\text{far}}\right\rangle },$$

where $\rho_{\text{NF},0}=\langle {C_{\text{rx}}}^{\;\text{near}}B_{1,\text{body}}^{+,\text{near}}\rangle /\langle {C_{\text{rx}}}^{\;\text{far}}B_{1,\text{body}}^{+,\text{far}}\rangle$ is a constant baseline and $C=\langle {C_{\text{rx}}}^{\;\text{near}}\rangle /\langle {C_{\text{rx}}}^{\;\text{far}}\rangle$.

When the wireless resonator is detuned ($\Delta B_{1,\text{wc}}^{+}=0$), $\rho_{\text{NF}}=\rho_{\text{NF},0}$ is constant across $\alpha_{\text{nom}}$; when it remains resonant below threshold, $\rho_{\text{NF}}$ rises above $\rho_{\text{NF},0}$. The $\rho_{\text{NF}}$‐versus‐$\alpha_{\text{nom}}$ curve is therefore L‐shaped, with the inflection point marking the detuning threshold. The perturbation is further amplified by C, which exceeds unity when the wireless resonator also enhances receive sensitivity—making the threshold visually distinct.

## 3 Methods

### 3.1 7T Experiments

To validate the proposed method, we constructed wireless resonators with different passive detuning voltage thresholds. Each resonator was fabricated from coaxial capacitors (COCA) [36, 37] and incorporated a frequency‐tuning network and a diode‐based passive detuning circuit (Figure 2A). The tuning and detuning components were housed in a small 3D‐printed enclosure lined with copper foil, which provided RF shielding to improve the stability and reproducibility of the detuning response. The design resembles the coaxial cable coil [38, 39, 40] but does not use its self‐resonance mode; the coaxial structure serves solely as a distributed capacitor [36, 37]. The resonators had a diameter of 5cm and were tuned to 298MHz, the Larmor frequency of our 7 T scanner. Nonmagnetic ceramic capacitors and crossed diodes of the same type used in our previous wireless resonator work were employed [22]. To vary the detuning threshold, we built two additional resonators with two and three pairs of crossed diodes, respectively; together with the original single‐pair resonator, these three resonators require progressively higher forward voltages to activate detuning. These configurations were used as a controlled methodological comparison rather than as practical design recommendations. In practice, increasing the number of series‐connected crossed‐diode pairs raises the detuning threshold and therefore limits the minimum usable flip angle.

Experiments were performed on a Philips Achieva whole‐body scanner (Philips Healthcare, Best, The Netherlands) equipped with a Nova 8TR/32Rx head array (Nova Medical, Wilmington, MA, USA), using the default fixed RF shim for all acquisitions. Measurements were performed using both a water‐based tissue‐mimicking phantom, with a conductivity of approximately 0.5S m^−1^ and a relative permittivity of approximately 55, and an oil‐mixture bottle phantom (height 30 cm, diameter 15 cm). For both phantoms, each wireless resonator was positioned directly above the phantom using the same flip‐angle‐swept GRE protocol was applied. 2D GRE images were acquired at a series of nominal flip angles spanning 0° – 20°, with details listed in Table 1.

**TABLE 1:** Summary of experimental setups at 7 and 3T.

	7T Philips	3T Philips	3T GE
*Hardware*			
Scanner	Achieva	Ingenia Elition 3.0T X	Discovery MR750
Tx coil	Local 8Tx (Nova)	Body	Body
Rx coil	32‐ch head	32‐ch head	16‐ch head/neck
Wireless resonator	Single element	1×3 array	1×3 array
Phantom	Bottle, ⌀ 15cm× 30cm (oil mixture) Sphere, ⌀ 11cm (water phantom)	Sphere, ⌀ 17cm (system QA gel phantom)	Cylinder, ⌀ 14cm× 30cm (model 5342679‐2)
*Acquisition (2D axial GRE)*			
TR / TE (ms)	300 / 3	300 / 2.3	300 / 2.9
FOV	220×220 mm^2^	220×220 mm^2^	384×384 mm^2^ (cropped to 220×220 mm^2^)
In‐plane res.	2.8×2.8 mm^2^	3×3 mm^2^	3×3 mm^2^
Slice thickness	10mm	10mm	10mm
$B_{1}^{+}$ @ 90°	18μT	13.5μT	7.3μT
Flip‐angle range	0°–20°	0°–20°	0°–20°
Flip‐angle step	1‐pair: 0.15° (<3°), 1° (≥ 3°) 2‐pair: 0.3° (<6°), 1° (≥ 6°) 3‐pair: 0.2° (<9°), 1° (≥ 9°)	0.3° (<6°), 1° (≥ 6°)	0.2° (<3°), 1° (≥ 3°)
Near ROI (ellipse)	Major/Minor = 1.5/0.6 cm (oil and water phantom)	Major/Minor = 1.5/1 cm	Major/Minor = 3/1 cm
Far ROI (ellipse)	Major/Minor = 3/3 cm (oil phantom) Major/Minor = 1.5/0.6 cm (water phantom)	Major/Minor = 1.5/1 cm	Major/Minor = 3/1 cm

### 3.2 3T Experiments

The wireless resonator used at 3T was a previously reported three‐element decoupled array (Figure 2B), adapted from the 1.5T design of Lu et al. [27]. Briefly, the array consists of three loops in a single row, with adjacent loops overlapped for geometric decoupling and nonadjacent loops decoupled via a bridging capacitor network. Each loop incorporates a passive detuning circuit comprising an add‐on inductor and a single pair of crossed diodes. The only modification relative to [27] was retuning the resonators to 128MHz (the Larmor frequency at 3T) by adjusting the capacitor values. The fabricated array measured 10×10 cm^2^ and is also shown in Figure 2B. The tuning and detuning components in the 3 T resonators were chosen from the same class as those used in [22] and housed in an RF‐shielded enclosure.

The array was tested on a Philips 3T scanner and a GE 3T scanner, placed above phantoms used for system QA. A flip‐angle sweep protocol similar to that in Section 3.1 was applied, with minor adjustments. Details of the setup are listed in Table 1. The two platforms use different reference $B_{1}^{+}$ values, which determine the RF pulse amplitude corresponding to a given nominal flip angle: 13.5μT on the Philips scanner (its default, such that $\alpha_{\text{nom}}=90$° corresponds to $B_{1}^{+}$ = 13.5μT), and 7.32μT on the GE scanner, derived from the transmit‐power calibration and read directly from the pulse‐sequence parameters for our setup.

### 3.3 Bench Characterization

Figure 2C shows a photograph of the benchtop setup used to characterize resonance and detuning performance for all wireless resonators in this study. A custom‐built double probe connected to a Keysight E5071C vector network analyzer (VNA) was inductively coupled to the resonator under test to measure its $S_{21}$ response. The double probe consisted of two circular loops, each 2cm in diameter, with a center‐to‐center spacing of 1.6cm. The measured $S_{21}$ response was used to determine the resonance frequency and quality factor under unloaded and phantom‐loaded conditions. A Keithley 2231A DC power supply was connected across the crossed‐diode detuning circuit to apply a controlled forward bias, allowing the detuned‐state $S_{21}$ response to be confirmed independently of RF drive.

This setup was used identically for the 7 T single‐element resonator (Figure 2A) and the 3 T three‐element array (Figure 2B). For the 7 T resonator, detuning efficacy was further characterized as a function of applied DC voltage for configurations containing one, two, and three crossed‐diode pairs, to obtain independently verified detuning‐voltage thresholds for each configuration.

### 3.4 Data Analysis

The same analysis was applied to all acquisitions. Two elliptical ROIs of identical size (details of the ROIs are listed in Table  1) were defined for each experiment: a *near* ROI adjacent to the wireless resonator, and a *far* ROI in a region where the resonator's influence is negligible. For each flip angle, we computed the spatial signal ratio $\rho_{\text{NF}}$ based on Equation (4).

Based on the theory in Section 2, a relatively constant $\rho_{\text{NF}}$ across the sweep indicates reliable passive detuning, whereas an elevated $\rho_{\text{NF}}$ at low flip angles indicates that detuning was not achieved. The turning point of the resulting L‐shaped curve marks the detuning threshold. The detuned‐state baseline $\rho_{\text{NF},0}$ was estimated by fitting a straight line to the large‐angle plateau region ($16^{\circ }$–$20^{\circ }$) and extrapolating across the full sweep range. We define the detuning threshold as the nominal flip angle at which $\rho_{\text{NF}}$ first exceeds $\rho_{\text{NF},0}$ by more than 10%, an empirical operational criterion chosen to provide a conservative margin above baseline noise and fitting uncertainty, rather than a value with intrinsic physical significance. The same criterion was applied consistently across all experiments.

## 4 Results

### 4.1 Bench Characterization

**FIGURE 3:**
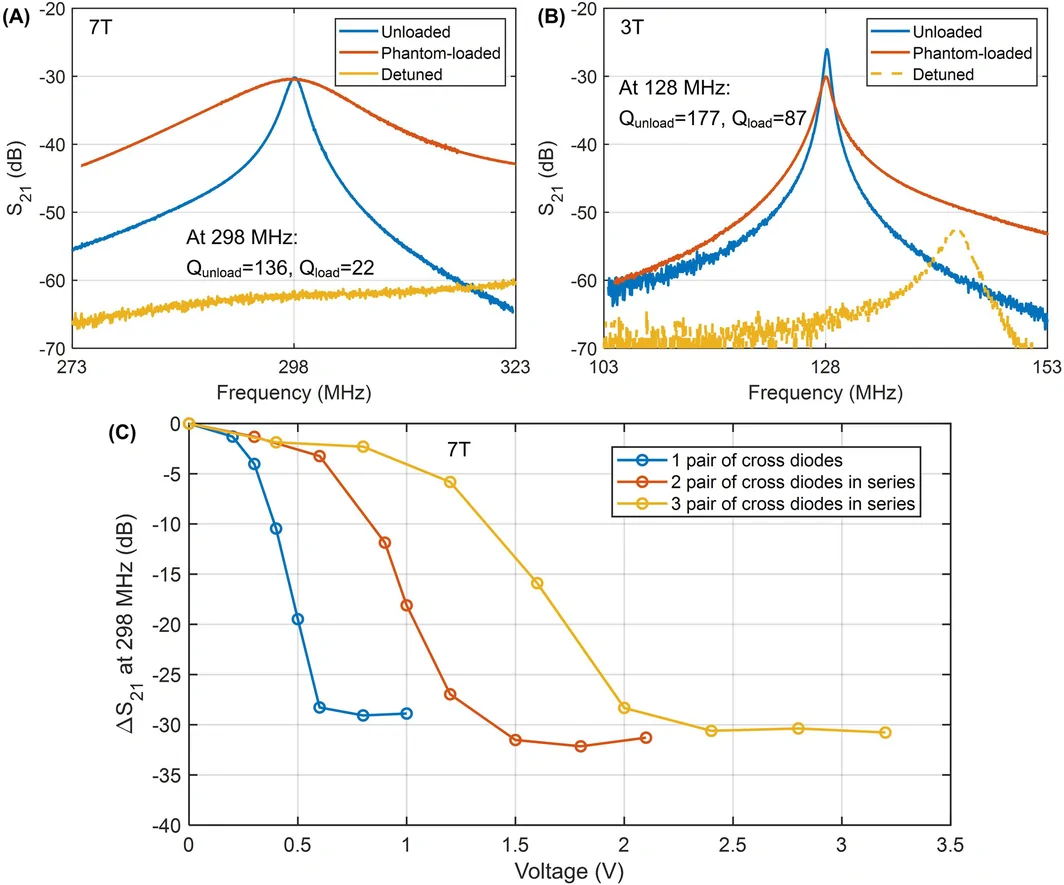
Benchtop characterization of the wireless resonators. (A) Double‐probe $S_{21}$ measurements of the 7 T single‐element wireless resonator under unloaded, phantom‐loaded, and detuned conditions. The unloaded and phantom‐loaded Q‐factors at 298 MHz were 136 and 22, respectively. (B) Double‐probe $S_{21}$ measurements of the 3 T three‐element wireless resonator array under unloaded, phantom‐loaded, and detuned conditions. The unloaded and phantom‐loaded Q‐factors at 128 MHz were 177 and 87, respectively. (C) Change in $S_{21}$ at 298 MHz as a function of applied DC bias voltage for the 7 T resonators containing one, two, or three crossed‐diode pairs connected in series. Increasing the number of crossed‐diode pairs shifted the detuning transition to a higher applied voltage.

Figure 3A,B show the measured $S_{21}$ responses of the 7 T single‐element resonator and the 3 T three‐element array, respectively. The resonance peaks were centered at the corresponding Larmor frequencies of 298 MHz at 7 T and 128 MHz at 3 T, confirming proper tuning. At 298 MHz, the unloaded and phantom‐loaded quality factors of the 7 T resonator were $Q_{\text{unload}}=136$ and $Q_{\text{load}}=22$, respectively. At 128 MHz, the corresponding values for the 3 T array were $Q_{\text{unload}}=177$ and $Q_{\text{load}}=87$. The 3 T array exhibited a single resonance peak without observable mode splitting, indicating effective inter‐element decoupling. At 128 MHz, the difference between the resonant and detuned $S_{21}$ responses was approximately 35 dB, demonstrating effective detuning of the 3 T array.

The detuning performance of the 7 T resonators was further characterized by measuring the change in $S_{21}$ at 298 MHz as a function of the applied DC voltage (Figure 3C). The approximate detuning‐voltage thresholds were 0.6, 1.5, and 2.4V for resonators containing one, two, and three crossed‐diode pairs, respectively. Increasing the number of crossed‐diode pairs shifted the detuning transition toward a higher applied voltage, consistent with the corresponding shifts in the $\rho_{\text{NF}}$ deviation toward higher flip angles.

### 4.2 Validation Across Detuning Thresholds at 7T

**FIGURE 4:**
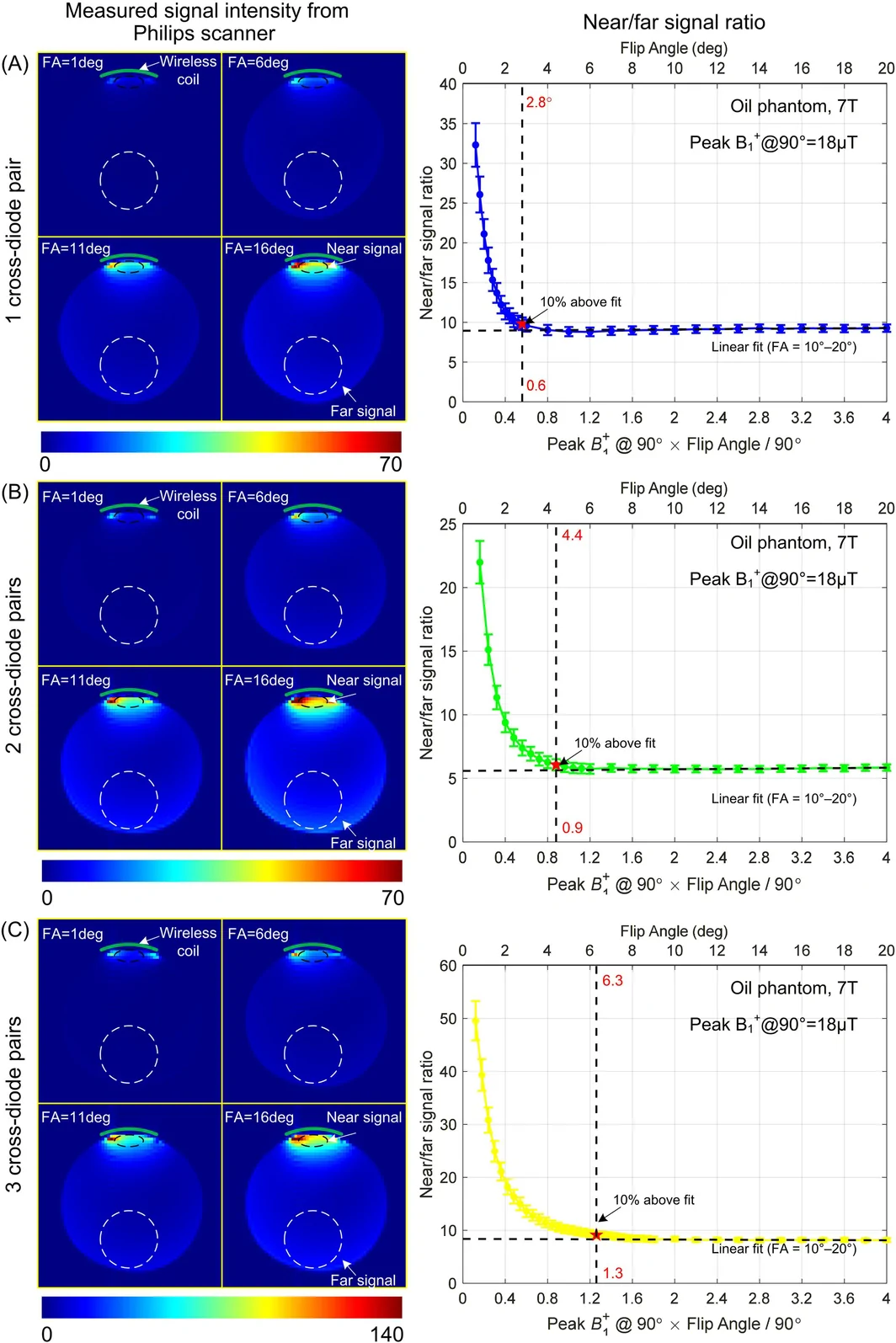
Validation of the passive‐detuning threshold measurement at 7 T using an oil phantom. (A–C) Representative GRE images and near‐to‐far signal ratio, $\rho_{\text{NF}}$, for 5‐cm‐diameter wireless resonators containing one, two, and three crossed‐diode pairs, respectively. Images acquired at flip angles of $1^{\circ },\;6^{\circ },\;11^{\circ }$, and $16^{\circ }$ are shown, with dashed ellipses indicating the near and far ROIs. The baseline was fitted over the $10^{\circ }$–$20^{\circ }$ range, and the threshold was defined as the first flip angle at which $\rho_{\text{NF}}$ exceeded the fitted baseline by more than 10%. The measured thresholds were $2.8^{\circ },\;4.4^{\circ }$, and $6.3^{\circ }$, corresponding to peak $B_{1}^{+}$ values of 0.6, 0.9, and 1.3μT, respectively. Error bars represent propagated ROI uncertainty; at higher flip angles, some are smaller than the plotted symbols.

Figure 4 presents representative 7 T GRE images and $\rho_{\text{NF}}(\alpha_{\text{nom}})$ curves acquired using the oil phantom at a reference $B_{1}^{+}$ of 18μT. For the resonators containing one, two, and three crossed‐diode pairs (Figure 4A–C, respectively), $\rho_{\text{NF}}(\alpha_{\text{nom}})$ remained close to the fitted baseline at higher flip angles, consistent with effective passive detuning, but increased markedly at lower flip angles as the induced voltage fell below the diode threshold. This transition occurs gradually over a finite flip‐angle range rather than as an abrupt step, because the diode current and dynamic resistance change continuously through the turn‐on region, while under RF excitation the conduction interval increases progressively with drive amplitude; consequently, the detuning‐circuit impedance, and therefore the resonator's response at the Larmor frequency, changes gradually rather than abruptly. This behavior is representative of small‐signal silicon p–n diodes generally [41],rather than specific to the particular diode used here, and is consistent with the similarly gradual change in $\Delta S_{21}$ at 298 MHz observed independently in the bench‐test data (Figure 3C). The measured detuning thresholds were approximately 2.8°, 4.4°, and 6.3°, respectively. Increasing the number of crossed‐diode pairs raised the forward‐voltage requirement and shifted the detuning threshold to a higher flip angle.

In the control experiment performed using the Nova head coil alone (Figure S2), the peripheral‐to‐center, left‐to‐right, and top‐to‐bottom signal ratios showed only slight gradual variation over the full flip‐angle range and no characteristic L‐shaped transition. In contrast, the near‐to‐far signal ratio measured with the wireless resonator present showed a pronounced increase at low flip angles. These findings support that the observed L‐shaped behavior is associated with incomplete passive detuning of the wireless resonator rather than intrinsic instability of the spatial signal‐ratio measurement.

**FIGURE 5:**
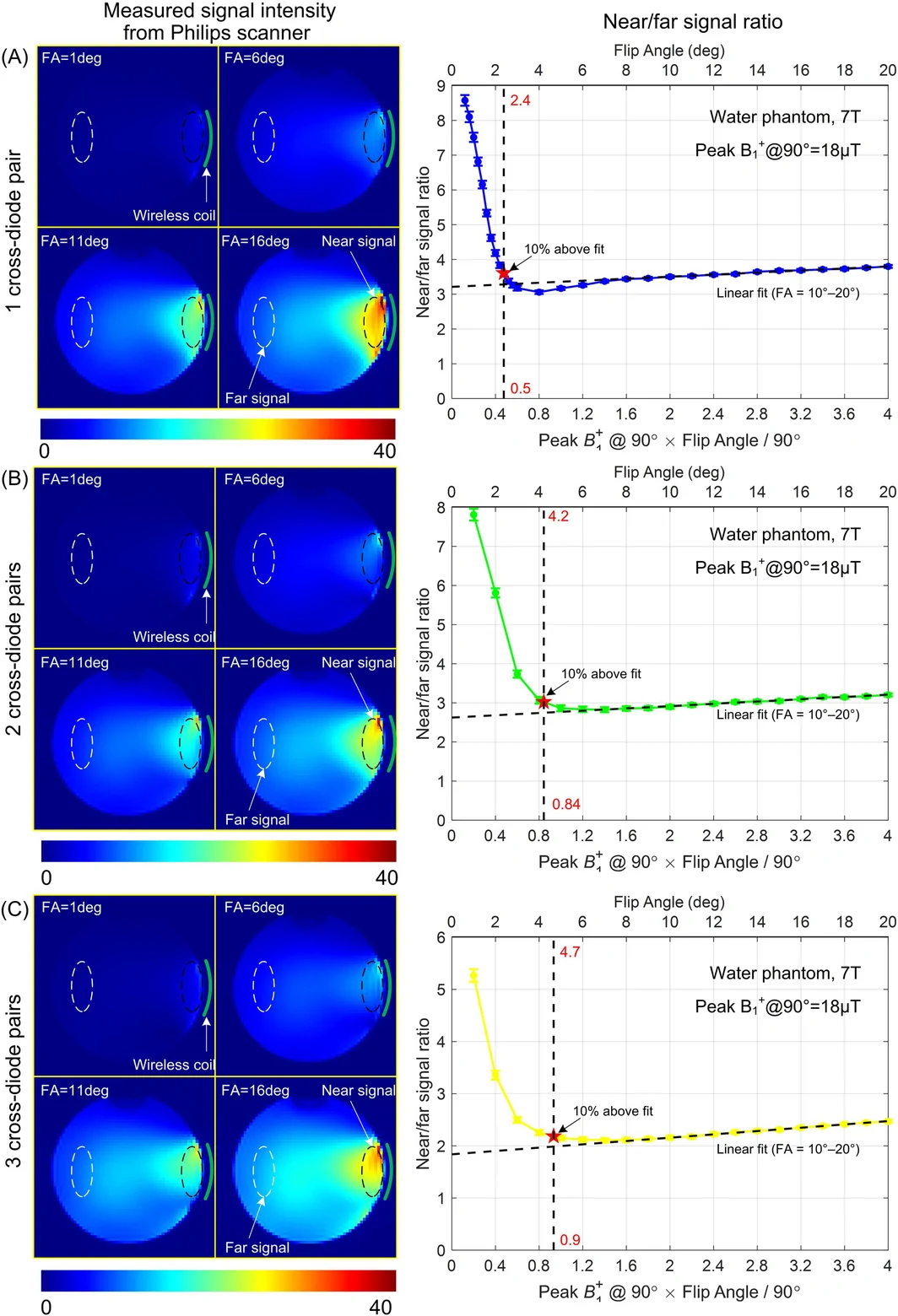
Validation of the passive‐detuning threshold measurement at 7 T using a water‐based tissue‐mimicking phantom. (A–C) Representative GRE images and near‐to‐far signal ratio, $\rho_{\text{NF}}$, for 5‐cm‐diameter wireless resonators containing one, two, and three crossed‐diode pairs, respectively. Images acquired at flip angles of $1^{\circ },\;6^{\circ },\;11^{\circ }$, and $16^{\circ }$ are shown, with dashed ellipses indicating the near and far ROIs. The baseline was fitted over the $10^{\circ }$–$20^{\circ }$ range, and the threshold was defined as the first flip angle at which $\rho_{\text{NF}}$ exceeded the fitted baseline by more than 10%. The measured thresholds were $2.4^{\circ },\;4.2^{\circ }$, and $4.7^{\circ }$, corresponding to peak $B_{1}^{+}$ values of approximately 0.5, 0.84, and 0.9μT, respectively. Error bars represent propagated ROI uncertainty; at higher flip angles, some are smaller than the plotted symbols.

The corresponding measurements using the water‐based tissue‐mimicking phantom are shown in Figure 5. The measured thresholds approximately were 2.4°, 4.2°, and 4.7° for the one‐, two‐, and three‐diode‐pair resonators, respectively. Although the absolute thresholds differed slightly between the two phantoms, both datasets showed the same systematic increase in detuning threshold with the number of crossed‐diode pairs, confirming that the proposed method consistently distinguishes resonators with different passive‐detuning voltage requirements. Importantly, the characteristic threshold behavior remained identifiable under the more tissue‐relevant dielectric and conductive loading of the water‐based phantom despite the increased field nonuniformity at 7 T.

### 4.3 Measured Detuning Thresholds and Validation at 3T

**FIGURE 6:**
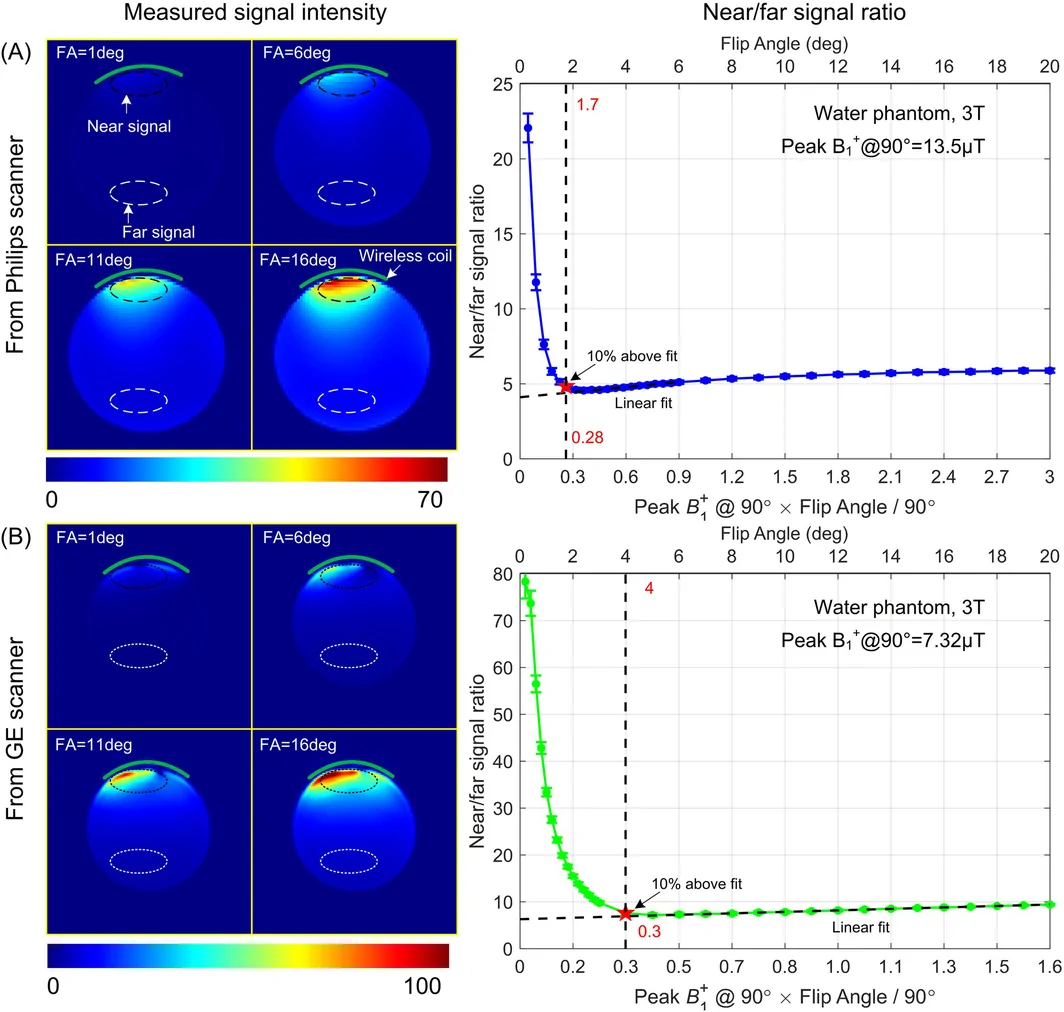
Validation of the passive‐detuning threshold measurement at 3 T using a water phantom. (A) Representative GRE images and near‐to‐far signal ratio, $\rho_{\text{NF}}$, acquired on the Philips 3 T scanner with a reference peak $B_{1}^{+}$ of 13.5μT. (B) Corresponding measurements acquired on the GE 3 T scanner with a reference peak $B_{1}^{+}$ of 7.32μT. Images acquired at flip angles of $1^{\circ },\;6^{\circ },\;11^{\circ }$, and $16^{\circ }$ are shown, with dashed ellipses indicating the near and far ROIs. The detuning threshold was defined as the first flip angle at which $\rho_{\text{NF}}$ exceeded the fitted baseline by more than 10%. The measured thresholds were approximately $1.7^{\circ }$ and $4.0^{\circ }$ on the Philips and GE scanners, respectively, corresponding to peak $B_{1}^{+}$ values of approximately 0.28μT and 0.30μT. Error bars represent propagated ROI uncertainty; at higher flip angles, some are smaller than the plotted symbols.

Figure 6 presents example GRE images and plots of $\rho_{\text{NF}}(\alpha_{\text{nom}})$ acquired on the Philips and GE 3T scanners. Consistent with the 7T results, $\rho_{\text{NF}}$ exhibited the same L‐shaped trend—remaining approximately constant at higher flip angles and rising at lower ones, once the induced voltage fell below the diode threshold.

Since the pulse sequences used on the Philips and GE scanners have different peak $B_{1}^{+}$ amplitudes, the measured flip‐angle thresholds differ between the two systems. For the Philips scanner with a reference $B_{1}^{+}$ of 13.5μT (Figure 6), the measured flip‐angle threshold is approximately 1.7°, whereas it shifted to approximately 4° for the GE scanner where the reference $B_{1}^{+}$ is 7.3μT. This is consistent with the expectation that a lower reference $B_{1}^{+}$ produces a lower absolute RF amplitude for any given nominal flip angle, so that a higher nominal flip angle is needed to generate sufficient voltage across the diodes. However, the corresponding $B_{1}^{+}$ threshold values for the two scanners are similar (0.28μT for Philips 3T and 0.3μT for GE 3T). This confirms the relationship between the detuning threshold and the reference $B_{1}^{+}$, and provides additional validation that the proposed method is genuinely measuring the onset of passive detuning failure.

### 4.4 Summary of Measured Thresholds

Across all experimental configurations tested in this study at 3 and 7 T, covering different wireless resonator geometries and numbers of resonator elements, the measured detuning flip‐angle threshold with a single pair of crossed diodes consistently remained below 5° under the default peak $B_{1}^{+}$ setting.

## 5 Discussion

### 5.1 Summary

We have presented a simple approach for quantifying the Tx‐power threshold above which passive detuning of wireless MRI receive resonators becomes reliable, based on the near/far spatial signal ratio from a single flip‐angle‐swept GRE acquisition. Although the underlying mechanism of passive detuning is straightforward, its behavior in practice depends on multiple factors: the pulse‐sequence amplitude and shape, the resonator components (especially the crossed diodes), and the mutual coupling between the Tx coil and the wireless resonator. The proposed method provides a phantom‐based way to evaluate when passive detuning becomes effective, independent of these design specifics.

At 7T, the method confirmed that the detuning flip‐angle threshold increases with the required total forward voltage, here controlled by the number of crossed‐diode pairs (one, two, and three). At 3T, it yielded consistent thresholds across the Philips and GE platforms, demonstrating cross‐vendor reproducibility.

### 5.2 Complementarity With $B_{1}^{+}$ Mapping

We emphasize that $B_{1}^{+}$ mapping is not performed in this study and is discussed here only as an existing, complementary technique. A flat $\rho_{\text{NF}}(\alpha_{\text{nom}})$ alone does not distinguish between two scenarios: (a) the detuning circuit is fully engaged at all tested flip angles (the resonator is always detuned), or (b) the detuning circuit is never engaged (the resonator is always resonant). However, in scenario (b), the $B_{1}^{+}$ perturbation from the wireless resonator would be readily detectable by a standard $B_{1}^{+}$ map acquired with and without the wireless resonator. The proposed method is therefore complementary to $B_{1}^{+}$ mapping: it addresses the case where $B_{1}^{+}$ maps with and without the wireless resonator are indistinguishable at moderate‐to‐high flip angles, and the remaining question is whether detuning also holds at the much lower transmit power levels that $B_{1}^{+}$ mapping cannot reliably probe.

### 5.3 Origin of the Gradual Tuned‐to‐Detuned Transition

The gradual transition might be attributed to the characteristics of the anti‐parallel switching diodes used in the detuning circuit, whose impedance changes continuously with drive rather than switching at a fixed voltage. The diodes require no external bias and are turned on by the current induced in the resonator itself. Because the forward current of a silicon junction rises exponentially with applied voltage, the differential resistance falls smoothly, with no discontinuity at the nominal turn‐on voltage. Under RF drive, the diodes conduct only during the fraction of each cycle in which the instantaneous voltage exceeds the junction potential, and this conduction angle widens progressively as the transmit amplitude increases, so the cycle‐averaged impedance of the detuning circuit decreases over a finite range of drive.

### 5.4 Practical Considerations for Scan Parameters

The method does not require high spatial resolution, since one can use mean signal within large ROIs rather than voxel‐wise mapping. Low resolution (large voxel size) is therefore not only acceptable but beneficial, as it improves SNR per voxel—particularly useful at the very low flip angles where SNR is inherently poor. Increasing slice thickness and signal averaging further improve SNR in this regime. If SNR remains insufficient at the lowest flip angles, the noise floor can be subtracted before applying the analysis. Estimating the noise standard deviation σ from a signal‐free region and using the corrected signal $\tilde{S}=\sqrt{S^{2}-\sigma^{2}}$ mitigates the positive bias introduced by the Rician noise distribution in magnitude images [42], which would otherwise inflate the signal at low flip angles and distort the ratio.

Total scan time depends on TR, the number of flip‐angle steps, and the sweep range. Our validation experiments used TR = 300ms with fine flip‐angle sampling (step size 0.15°–0.3° near the threshold) to characterize the method on a dense dataset. For routine use, neither the dense sampling nor the long TR is required: coarser sampling centered on the expected threshold, combined with a shorter TR, can bring the total acquisition down to roughly 2–3 min.

### 5.5 Generalizability

The method is general and applicable wherever wireless resonators are used. Its only requirements are a phantom and a flip‐angle‐swept GRE sequence—both universally available on clinical and research scanners. It is also agnostic to the specific passive detuning topology (e.g., crossed diodes shorting or opening a parallel capacitor), because it detects the functional outcome—the $B_{1}^{+}$ (or equivalently, the flip angle for a given peak $B_{1}^{+}$) at which the wireless resonator begins to perturb the transmit field—rather than the circuit mechanism. Validation across 3T and 7T and across Philips and GE platforms demonstrates this vendor‐ and field‐strength independence.

### 5.6 Phantom‐Based Validation and Translation to In Vivo Use

Phantom‐based testing is not a limitation of this method but its necessary setting: the measurement deliberately probes the low‐flip‐angle regime in which the resonator is not yet fully detuned, a condition that cannot be entered intentionally in a human subject.

Since the threshold also depends on the setup, in particular the orientation of the resonator relative to the transmit coil and the loading condition, this dependence might raise concern when phantom results are applied to patients. The dependence is, however, partly self‐compensating. A weakly coupled orientation raises the threshold, but in that same configuration the resonator's ability to perturb the $B_{1}^{+}$ and electric fields, should it fail to detune, is correspondingly smaller. Heavier loading raises the equivalent resistance and reduces the induced current, but also demands higher transmit power for a given flip angle, so the threshold expressed in flip angle changes little. Consistent with this, our oil‐ and water‐phantom experiments at 7 T, representing two extremes of loading, showed only modest differences in threshold.

We nonetheless recommend that the test be performed with the resonator in a similar position and orientation to that used in vivo, as was done here, and with a tissue‐mimicking phantom. A safety margin should also be applied, and establishing an appropriate margin factor is left to future work.

### 5.7 Preliminary Evaluation at 0.55T

The wireless resonator designs used here are well established at 3 and 7 T, where the detuning threshold is low under the scanner's default peak $B_{1}^{+}$ setting. Motivated by renewed interest in mid/low‐field systems such as 0.55T [43], we performed a preliminary translation study by building a simple 4‐element wireless coil array and repeating the same detuning evaluation. Using the scanner's default peak $B_{1}^{+}$ (11.2 μT), we observed a detuning threshold of approximately 9°, substantially higher than the $<5\overset{ˇ}{r}$ thresholds measured at 3 and 7 T (Figure S1). This increase is expected because, for a fixed reference $B_{1}^{+}$, the RF current induced in the resonator scales with frequency; thus, the same diode forward‐voltage threshold corresponds to a higher flip angle at lower field strength. Although this 0.55T implementation is not yet optimized, two practical strategies could reduce the threshold. First, using shorter RF pulse durations (if supported by the scanner) would increase peak $B_{1}^{+}$ at a fixed nominal flip angle, thereby promoting detuning at lower angles. Second, multi‐turn resonator designs are particularly attractive at low field and should outperform the single‐turn geometry used here by increasing mutual coupling to the body transmit coil and enabling a smaller parallel capacitance across the crossed‐diode circuit; both effects raise the RF voltage across the diodes for a given transmit power and lower the effective detuning threshold.

### 5.8 Other Passive Resonators

The method extends naturally beyond wireless loop and birdcage‐type resonators to other receive‐only passive resonant structures, including metamaterial and metasurface resonators [44, 45, 46, 47, 48]. These structures interact with the transmit field through the same mechanism: when properly detuned they are transparent to $B_{1}^{+}$; when detuning fails they perturb $B_{1}^{+}$ locally. Because our method detects this functional outcome rather than probing the internal circuit topology, it applies directly to any passive insert that relies on nonlinear switching elements for detuning.

## 6 Conclusion

We have introduced a simple spatial signal ratio method for evaluating the Tx‐power threshold required for reliable passive detuning of wireless MRI receive resonators. It requires only a standard GRE sequence and a homogeneous phantom, and needs no $B_{1}^{+}$ mapping or control scan. Experimental validation with wireless resonators incorporating one, two, and three pairs of crossed diodes at 7T, and with arrays at 3T (Philips and GE), confirmed that the method correctly identifies and distinguishes detuning thresholds. With a single crossed‐diode pair, the measured threshold remained below 5° at 3 and 7T—well below the flip angles used in most pulse sequences.The method is straightforward, vendor‐ and field‐independent, and requires no specialized hardware or software, making it a practical tool for acceptance testing and quality assurance of wireless resonator safety in clinical and research MRI.

## Author Contributions

**Ming Lu:** investigation, formal analysis, software, visualization. **Xiaoyu Jiang:** investigation, data curation, formal analysis. **Yajun Ma:** investigation, data curation, formal analysis. **Yurui Qian:** investigation, data curation. **Congyu Liao:** investigation, data curation. **Yang Yang:** investigation, data curation. **John C. Gore:** supervision. **Xinqiang Yan:** conceptualization, methodology, formal analysis, writing – original draft, supervision, funding acquisition. All authors contributed to writing – review and editing and approved the final manuscript.

## Funding

This work was supported in part by the Vanderbilt Innovation Catalyst Fund and NIH grants R01 EB031078 and S10 OD030389.

## Conflicts of Interest

The authors declare no conflicts of interest.

## Supporting information

Supplementary Information1.docx.

## Data Availability

The data that support the findings of this study are available from the corresponding author upon reasonable request.
