# Supplementary material for: Quantifying the Transmit‐Power Threshold for Reliable Passive Detuning of Inductively Coupled Wireless Resonators in MRI: A Phantom Study
Source: NMR Biomed. 2026 Sep 25;39(11):e70408. doi: 10.1002/nbm.70408 (PMC13613141; doi:10.1002/nbm.70408)
Supplement: Supplementary file 1 [file NBM-39-e70408-s001.docx]

**Supplementary Materials**

The Supplementary Information includes 2 Supplementary Figures.

**Quantifying the Transmit-Power Threshold for Reliable Passive Detuning of Inductively Coupled Wireless Resonators in MRI: A Phantom Study**

*Ming Lu¹, Xiaoyu Jiang¹˒², Yajun Ma³, Yurui Qian⁴, Congyu Liao⁴, Yang Yang⁴, John C. Gore¹˒², Xinqiang Yan¹˒²˒⁵˒**

¹Vanderbilt University Institute of Imaging Science, Vanderbilt University Medical Center, Nashville, TN, United States

²Department of Radiology and Radiological Sciences, Vanderbilt University Medical Center, Nashville, TN, United States

³Department of Radiology, University of California, San Diego, CA, United States

⁴Department of Radiology and Biomedical Imaging, University of California, San Francisco, CA, United States

⁵Department of Electrical and Computer Engineering, Vanderbilt University, Nashville, TN, United States

**Corresponding Author (**†**):**

Xinqiang Yan, Ph.D.

Vanderbilt University Institute of Imaging Science

1161 21st Avenue South
Medical Center North, D-2205
Nashville, TN 37232-2310

Phone No: 1(615) 5253989

Email: [xinqiang.yan@vumc.org](mailto:xinqiang.yan@vumc.org)

**Figure S1**	(A) Circuit diagram of the wireless resonator designed for Siemens 0.55 T MRI. (B) Layout of the wireless resonator array showing geometric overlap for inter-element decoupling. (C) Measured signal intensity from the Siemens 0.55 T scanner at different flip angles. (D) Near/far signal-intensity ratio R as a function of flip angle and corresponding B_1_^+^ value. The ratio R remains constant above the detuning threshold and becomes elevated below it, forming the characteristic L-shaped curve. The threshold was determined as the point where R reached 10% above the high-flip-angle linear fit.

**Figure S2**.	Control experiment confirming the intrinsic stability of the near-to-far signal ratio in the absence of the wireless resonator. Left: representative axial signal-intensity maps of the Nova head coil alone (no wireless resonator present) at nominal flip angles of 10° and 20°, showing the regions of interest used to compute reference signal ratios: peripheral, center, top, bottom, left, and right. Right: signal ratio as a function of nominal flip angle (top axis) and peak $B_{1}^{+}$at 90° scaled by flip angle/90° (bottom axis, a normalized measure of local transmit amplitude). The purple curve shows the near-to-far signal ratio, $\rho_{\mathrm{NF}}$, measured with the wireless resonator present, exhibiting the characteristic L-shaped rise at low flip angles described in the main text. The yellow, red, and blue curves show the corresponding peripheral/center, left/right, and top/bottom signal ratios of the Nova head coil alone, with the wireless resonator absent; these remain approximately constant across the full flip-angle range, confirming that the L-shaped behavior arises specifically from incomplete passive detuning of the wireless resonator rather than from intrinsic instability of the signal-ratio measurement itself.
